# Audience effects on the neural correlates of relational reasoning in adolescence

**DOI:** 10.1016/j.neuropsychologia.2016.05.001

**Published:** 2016-07-01

**Authors:** Iroise Dumontheil, Laura K. Wolf, Sarah-Jayne Blakemore

**Affiliations:** a UCL, Institute of Cognitive Neuroscience, 17 Queen Square, London WC1N 3AR, UK; b Department of Psychological Sciences, Birkbeck, University of London, Malet Street, London WC1E 7HX, UK

**Keywords:** Adolescence, Peer influence, FMRI, Audience effect, Reasoning

## Abstract

Adolescents are particularly sensitive to peer influence. This may partly be due to an increased salience of peers during adolescence. We investigated the effect of being observed by a peer on a cognitively challenging task, relational reasoning, which requires the evaluation and integration of multiple mental representations. Relational reasoning tasks engage a fronto-parietal network including the inferior parietal cortex, pre-supplementary motor area, dorsolateral and rostrolateral prefrontal cortices. Using functional magnetic resonance imaging (fMRI), peer audience effects on activation in this fronto-parietal network were compared in a group of 19 female mid-adolescents (aged 14–16 years) and 14 female adults (aged 23–28 years). Adolescent and adult relational reasoning accuracy was influenced by a peer audience as a function of task difficulty: the presence of a peer audience led to decreased accuracy in the complex, relational integration condition in both groups of participants. The fMRI results demonstrated that a peer audience differentially modulated activation in regions of the fronto-parietal network in adolescents and adults. Activation was increased in adolescents in the presence of a peer audience, while this was not the case in adults.

## Highlights

• We examined the effect of the presence of a peer audience on relational reasoning.
• Mid-adolescent and adult sensitivities to a peer audience were compared.
• Accuracy of relational integration was affected by a peer audience.
• Relational reasoning activation was modulated by a peer audience.
• The peer audience differently modulated brain activation in adolescents and adults.

## 1 Introduction

Adolescence is defined as the period of life that starts with puberty and ends when an individual attains a stable, independent role in society (Lerner and Steinberg, 2004). Substantial changes in the social environment occur during adolescence; in particular, adolescents’ relationships with peers become increasingly important (Brown, 2004). These environmental changes are thought to coincide with a heightened sensitivity to social contexts and thus influence adolescent behaviour (Blakemore and Mills, 2014).

The aim of the current functional magnetic resonance imaging (fMRI) study was to investigate developmental differences in the influence of a peer audience on the neural correlates of a high-level cognitive task between adolescence and adulthood. We assessed the influence of a peer audience on the activation of brain regions associated with relational reasoning, which is a type of fluid reasoning defined as the ability to think logically and solve problems independent of prior knowledge, and is associated with academic achievement (Ferrer et al., 2009, Krawczyk, 2012). Because of their greater sensitivity to social context, an adolescent asked to solve a mathematics or logic problem on the whiteboard in front of their peers or in a one-on-one situation with their tutor may be more affected by the presence of an audience than a child or adult in the same situation. This type of effect may be broadly categorised as “choking under pressure”, in this case social pressure (Belletier et al., 2015).

Tests such as the USA Law School Admission Test include sections (Logic Games, Logical Reasoning) that heavily tax relational reasoning (Mackey et al., 2012). Similarly, tests of non-verbal reasoning, which similarly tax relational reasoning, are often used in the selection process for academically selective schools in late childhood/early adolescence in the UK. If relational reasoning performance is affected by the presence of an audience (e.g. other students, known teachers or neutral invigilators), this may have consequences in terms of test outcomes and therefore future academic progression of pupils.

### 1.1 Heightened sensitivity to peer influence in adolescence

Compared with children, adolescents are more sensitive to peer influence (Brown, 2004, Steinberg and Silverberg, 1986) and are more concerned about being accepted by their peers (O’Brien and Bierman, 1988). Peer approval becomes increasingly important for self-esteem during adolescence relative to late childhood (O’Brien and Bierman, 1988). While fears about being punished by parents or teachers decrease between late childhood and adolescence (8–18 years), fears about being socially judged increase (Westenberg et al., 2004, Westenberg et al., 2007). These questionnaire-based studies suggest that adolescents are particularly concerned about being evaluated by their peers.

Experimental studies on peer influence have so far predominantly focussed on risky and reward-related decision-making and suggest that the presence of peers modulates adolescent reward sensitivity (Chein et al., 2011, Gardner and Steinberg, 2005, O’Brien et al., 2011, Reynolds et al., 2013, Weigard et al., 2014). Gardner and Steinberg (2005) showed that adolescents (13–16 years) made more risky decisions in a driving game when they were being observed by two peers compared to when they were on their own, while adult levels of risky decisions were not affected by the presence of peers (Gardner and Steinberg, 2005). A study employing an fMRI version of this driving game also showed developmental differences in the modulation of the activation in reward-related regions (the ventral striatum and the orbitofrontal cortex) in the presence of peers (Chein et al., 2011). In the decision period of the driving game, adolescents (14–18 years) activated these regions more in the presence of peers than when alone, while activation in these regions was not significantly affected by the presence of peers in young adults (19–22 years). The current fMRI study aimed to investigate whether this heightened adolescent sensitivity to peer influence also extends to differential peer audience effects on the neural system involved in a high-level cognitive task in adolescents and adults.

### 1.2 Audience effects

There is a long history of social psychology research (predominantly in adults) on social facilitation or, more specifically, the audience effect (Zajonc, 1965). The audience effect describes the influence of the presence of an audience on task performance, such as task accuracy, task speed or reaction time (RT) and has been studied with a range of different tasks and audiences. Audience effect studies have found that the presence of an audience is generally associated with performance improvement in simple tasks and performance impairment in complex or learning tasks, although there are inconsistencies in the literature due to the variety of tasks, methods and audience conditions employed (Aiello and Douthitt, 2001, Belletier et al., 2015, Bond and Titus, 1983, Zajonc, 1965). Two processes have been proposed to underlie impairments in performance. Task-irrelevant thoughts and worries may distract executive attention away from task execution, which leads to poorer performance when tasks require attentional control. Alternately, the desire to do well may lead to too much executive attention being directed to the task at hand, which may cause poorer performance on simple tasks that rely on skills and processes that are automatic and run best outside of conscious awareness (see Belletier et al. (2015) for a review).

A small number of studies have investigated the neural basis of the audience effect. A near-infrared spectroscopy (NIRS) study investigated the effect of an evaluative audience of two experimenters in a competitive scenario on an n-back working memory task. In the social context (competitive audience condition), participants made more errors than when they were alone in the most difficult n-back condition (3-back), and this behavioural difference was correlated with heightened activation in the prefrontal cortex in the 3-back condition compared to a baseline task. However, as the audience condition of that study included a competitive component, it is not clear whether this effect is attributable to the audience, to the competition or to both. A recent fMRI study investigated the audience effect on a motor task (Yoshie et al., 2016). When being observed performing a grip force task, participants generated more grip force output and this was accompanied by reductions in activity in bilateral inferior parietal cortex, which was correlated with individual differences in the socially-induced change in grip force.

The lack of previous fMRI studies investigating the effect of a non-competitive audience on task-related activation in a *high-level cognitive task* prevented us from making clear predictions regarding the direction of the modulation of task-related activation by the presence of an audience. The presence of an audience might act as an attentional distractor. On the one hand, it has been suggested that attentional distractors induce compensatory mechanisms, leading to increased activation in task-related regions reflecting neural efforts to uphold the level of task performance (Wessa et al., 2013). On the other hand, there is evidence supporting the idea that a distractor diverts attention away from the task, resulting in decreased activation in task-related regions (Dolcos and McCarthy, 2006, Mitchell et al., 2008). The current study is a first investigation of the neural correlates of the audience effect during reasoning in adolescents and adults. As such, it was not clear *a priori* whether the presence of an audience would lead to increased or decreased activation in the relational reasoning network. Findings of increased activation in the relational reasoning network would suggest that the presence of an audience leads to increased activity to support task performance, possibly due to compensatory mechanisms, while decreased activation would support the idea that the presence of an audience diverts neural processing away from the task.

### 1.3 Study design

The current study investigated the peer audience effect on the neural correlates of relational reasoning. Solving relational reasoning problems requires the generation of abstract mental relationships of features in a puzzle (e.g. a change in size, number or shape), and the integration of those relationships. Relational reasoning involves a fronto-parietal network including the inferior parietal lobule (IPL), the pre-supplementary motor area (preSMA), the dorsolateral prefrontal cortex (DLPFC) and the rostrolateral prefrontal cortex (RLPFC), the latter region being specifically associated with relational integration (Christoff et al., 2001, Crone et al., 2009, Dumontheil et al., 2010, Krawczyk, 2012).

A recent fMRI study employed a minimal, virtual peer manipulation, in which participants were simply being told that a peer was watching via a camera while they were lying in the scanner. Contrasting this peer condition with an alone condition resulted in higher levels of reported embarrassment, as well as greater activation in the medial prefrontal cortex (mPFC) – a key region of the social brain (Frith and Frith, 2007) - in adolescents relative to children (Somerville et al., 2013). In addition, autonomic arousal levels, measured by skin conductance, were heightened in adolescents relative to both children and adults, suggesting that the presence of peers is particularly salient and arousing during adolescence, even when a minimal, virtual peer manipulation is employed.

The current study used a similar, minimal, virtual peer audience manipulation to investigate the effect of being observed and evaluated by an unfamiliar peer on activation within a functionally defined relational-integration neural network, in a group of mid-adolescents (14–16 years) and adults (23–28 years). We adapted a relational reasoning paradigm that has been employed in previous neuroimaging studies with adults and adolescents (Christoff et al., 2003, Dumontheil et al., 2010, Smith et al., 2007, Wendelken et al., 2011). The paradigm includes both a simple Control task, in which problems are solved by considering a single relation (one-relational problems) and a complex Relational task, in which two relations need to be jointly considered and integrated (two-relational problems). This allows a comparison between peer audience effects in simple and complex versions of the task.

Previous studies have indicated that there are gender differences in adolescent sensitivity to evaluation by others, and particularly by peers: adolescent girls report higher levels of public self-consciousness (Rankin et al., 2004), greater importance of peer approval for self-esteem (O’Brien and Bierman, 1988) and greater fear of negative evaluation by peers (La Greca and Lopez, 1998, La Greca and Stone, 1993, Rudolph and Conley, 2005), than boys. Furthermore, adolescent boys and girls show differences in their peer relationships (for a review see Rose and Rudolph, 2006). FMRI studies have also suggested developmental differences in functional activation patterns during the anticipation of peer evaluation in female and male adolescents (Guyer et al., 2009). Consequently, in order to reduce noise in the sample due to potential sex differences, and investigate the peer audience effect in a population who may be particularly susceptible to it, this study only included female participants.

### 1.4 Peer audience effects during adolescence

While the majority of experimental studies investigating peer influence in adolescence have focused on risky- or reward-related decision-making, we recently found that adolescents’, but not adults’, performance in a relational reasoning task was sensitive to a peer audience in a behavioural study (Wolf et al., 2015). This peer audience effect was more pronounced in mid-adolescents (14.9–17.8 years), with both accuracy and RT being compromised in the presence of a peer relative to a non-peer, while young adolescents (aged 10.6–14.2 years) were less accurate in the presence of a peer only in simple reasoning trials. This behavioural study suggests that adolescents’ heightened sensitivity to a peer audience is not limited to risky- and reward-related decision-making, but extends to high-level cognitive task performance.

The current study aimed to investigate developmental changes in the effect of a peer audience on the neural correlates of relational reasoning. The task was similar to that used in our behavioural study but was adapted for fMRI in several ways, including the following: an implied, virtual and unfamiliar observer was used, rather than a real observer who was known to the participant; the task only included level 1 and level 2 relational reasoning (relational integration) with a limited number of stimuli, rather than more varied levels of relational integration and more varied stimuli; and the inter-stimulus-interval was fixed rather than allowing self-paced responses. The aim of the study was to investigate both behavioural differences between conditions, and differences in the activity of the relational reasoning network, which was predicted to be affected by an audience, especially in adolescents.

With regard to behaviour, we first asked whether relational reasoning performance is affected when participants think they are being observed by a peer via a camera. Previous audience effect studies have shown differential audience effects for simple and complex tasks (Bond and Titus, 1983); consequently we were interested in whether peer audience effects might differ between the relational integration task and the control task. Second, we investigated whether there are also developmental differences in this peer audience effect when a minimal, virtual peer manipulation is used.

With regard to our neuroimaging analysis, we first anticipated that a fronto-parietal network would be activated in the relational integration task relative to the control task, as has been previously shown, and that there might be developmental changes in this network (for a review see Dumontheil, 2014). Second, we investigated whether a peer audience modulates the activation within this relational-integration network and whether this modulation differs between adolescents and adults. We conducted a voxel-wise analysis within the relational-integration network to identify task-related regions that are modulated by peer audience observation. Due to the absence of previous fMRI studies investigating the audience effect on high-level cognitive tasks, it was not clear whether the peer audience would lead to an increase or a decrease in activation in the relational-integration network, however we predicted that the effect would be more pronounced in adolescents than in adults. Third, we studied whether a peer audience modulates the activation of the relational-integration network differently for the manipulation of single relations (Control) to the integration of relations (Relational), and whether this differs between adolescents and adults.

In addition to broadening our knowledge about the extent of adolescent sensitivity to peer influence, i.e. whether a differential modulation of activation is also found for a high-level cognitive task-network, findings from this study might also have potential implications for education. Adolescents spend a large proportion of their time at school in the presence of peers. A better understanding of how peers influence adolescents’ performance in cognitive tasks might help to design environments which, on the one hand, facilitate the acquisition of critical skills and, on the other hand, allow adolescents to learn how to excel in tasks in the presence and under the evaluation of peers.

## 2 Methods

### 2.1 Participants

Twenty-three female mid-adolescents and 18 female adults participated in the study. Data from 19 mid-adolescent participants (aged 14.2–16.7 years, mean±SD =15.5±0.9) and 14 adult participants (aged 23.1–28.8 years, 24.8±1.4) were included in the final analysis (see Debriefing Section (2.5)). Adolescent participants were mostly recruited from selective schools in the Greater London area. Adult participants were students or graduates, recruited via advertisement at university. In order to maximise susceptibility to the peer influence manipulation and to match adolescent and adult participants, adults were not invited to participate if they had taken part in five or more psychology or neuroscience experiments, or if they were students or graduates of Psychology, Neuroscience or related subjects. Study procedures were approved by the local Research Ethics Committee. Adult participants and parents or legal guardians of adolescent participants gave their informed consent for the study. Participants were reimbursed £10 per hour for taking part in the study.

The verbal subtest of Wechsler Abbreviated Scale of Intelligence (WASI, Wechsler, 1999) was used to estimate participants’ verbal IQs. Adolescent (118.5±8.4) and adult (121.4±5.2) participants did not significantly differ in verbal IQs (p>0.25). Participants also completed the resistance to peer influence questionnaire (RPI, Steinberg and Monahan, 2007). Adolescents (2.97±0.22) and adults (3.08±0.22) did not significantly differ in their RPI scores (p>0.15).

### 2.2 Study design

The fMRI study employed a block design with two within-subjects factors: Task (Relational; Control) and Audience (Peer; Alone) and one between-subjects factor: Age group (Adolescent; Adult).

#### 2.2.1 Task factor

**Fig. 1:**
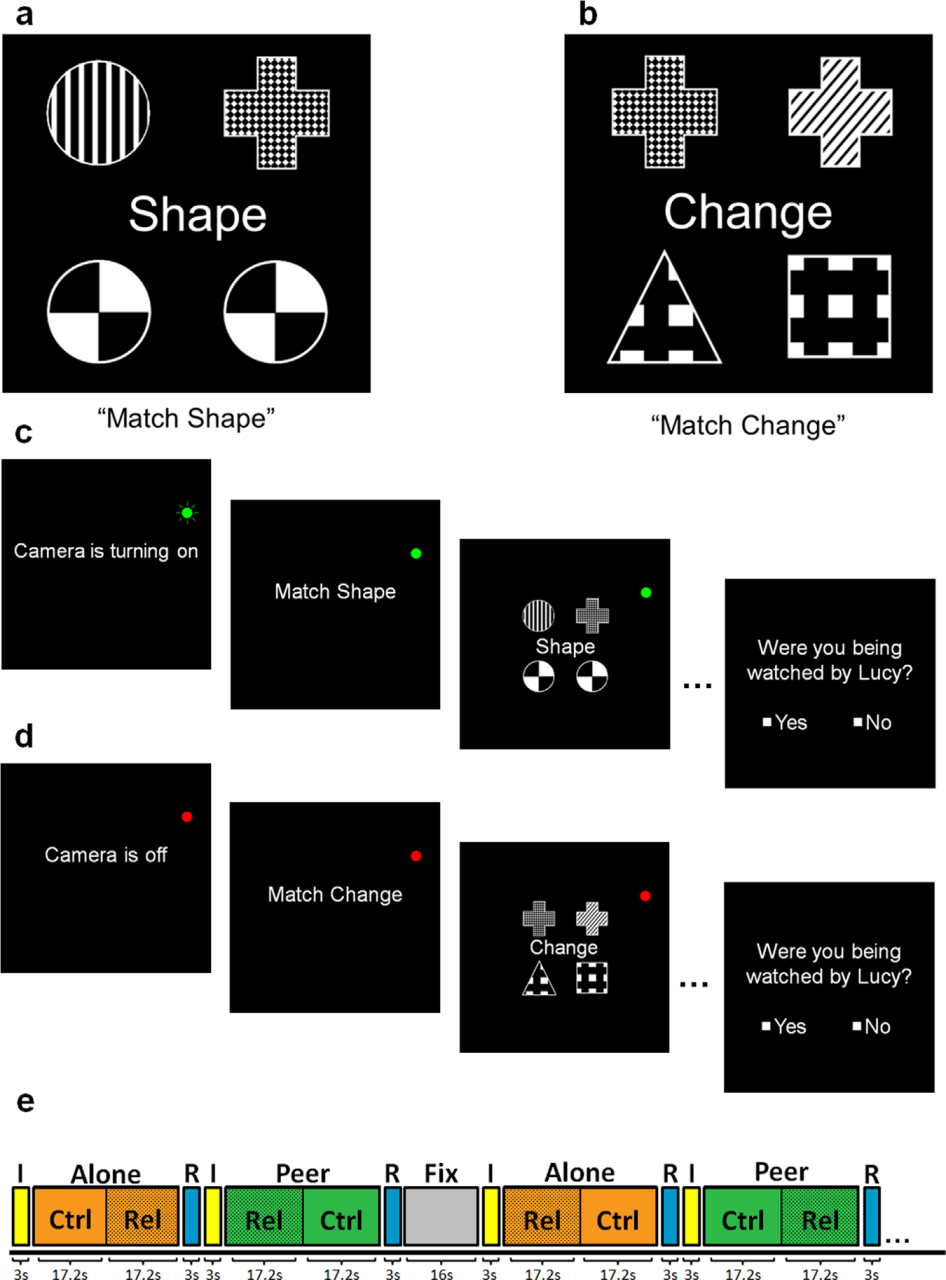
Relational reasoning task and peer audience manipulation. The instructions: ‘Shape’, ‘Pattern’ or ‘Change’ appeared in the middle of the screen in each trial to remind participants of the task they were performing. a) Example of a *Control* condition trial: participants were asked if either of the top two items were the same shape (or pattern) as the bottom two items. In this ‘Match Shape’ example, the top left item is the same shape (circle) as the bottom two items, thus the answer is ‘yes’. b) Example of a *Relational* condition trial: participants were asked if the top two items changed in the same way as the bottom two items. Here, the top pair differs in the ‘pattern’ dimension, while the bottom pair differs in the ‘shape’ dimension, thus the answer is ‘no’. **c)** Example of a *Peer* block: Prior to the Peer block, a screen along with a green, flashing light informed participants that the camera was turning on. Throughout the rest of the Peer block a green, constant light reminded participants that the camera was on. d) Example of an *Alone* block: Prior to the Alone block, a screen informed participants that the camera was off, along with a constant red light that was present throughout the Alone block. e) In each session, participants performed ten alternating Audience blocks (five *Peer* and five *Alone*). Prior to each Audience block, participants viewed an instruction screen (I in Figure) indicating whether the camera was turning on (Peer) or was off (Alone). Following an Audience block, participants were asked whether or not they had been observed (Camera rating, R in Figure). After every second Audience block there was a Fixation baseline block (*Fix*). Each Audience block contained one Control block (*Ctrl*, 4 trials) and one Relational block (*Rel*, 4 trials). Five Audience blocks started with a Control block and five with a Relational block; this was randomized within a session. (For interpretation of the references to color in this figure, the reader is referred to the web version of this article.)

The study employed a non-verbal relational reasoning task previously used by Dumontheil et al., (2010), adapted from Christoff et al., 2003, Smith et al., 2007). Methodological details can be seen in Fig. 1 and Dumontheil et al. (2010). Briefly, relational reasoning puzzles comprised two pairs of geometrical items aligned in a two-by-two grid. These items varied in shape (six different shapes) and pattern (six different patterns). In the Control condition, participants were asked whether the bottom two items (identical in this condition) *matched* either of the top two items along a specified dimension (shape or pattern) (Fig. 1(a)). In the Relational condition, participants were asked whether the top pair of items *varied* along the same dimension as the bottom pair of items (Fig. 1(b)). Task instructions were given at the beginning of a Task block (Control: ‘Match Shape’ or ‘Match Pattern’; Relational: ‘Match Change’) and in each trial a word cue in the middle of the screen reminded participants of the Task type. Participants entered ‘yes’ or ‘no’ responses with the index or middle finger of their right hand. Prior to scanning, participants were instructed on the task and trained to a criterion of 75% accuracy (all participants met this criterion after a few minutes of training (range: 1 min 46 s–4 min 21 s)).

#### 2.2.2 Audience factor

Before the experiment began, participants were told that an unfamiliar, similar-aged, same-sex peer would observe them and evaluate their performance at several points during the experiment via a camera mounted near the participant's face in the scanner. The peer was described as a work-experience student from a secondary school to the adolescent participants and as a junior post-graduate student to adult participants; the intention of this manipulation was to convince each participant that their performance would sometimes be observed by an unknown peer of around their own age. Prior to each Audience block, a screen indicated whether the camera was turning on (Peer condition, flashing green light, Fig. 1(c)) or the camera was off (Alone condition, constant red light, Fig. 1(d)). During each Audience block a constant green or red light reminded participants whether the camera was on or off. The participants were told that the peer would be observing them when the camera was on, but not when the camera was off. A similar camera manipulation has been previously used by Somerville et al. (2013). However, in addition to being told that the peer would be observing them when the camera was on, in our study participants were led to believe that the peer would also evaluate their performance when the camera was on. The camera was pointed out to participants while they were being prepared for the scanning session. To enhance the credibility of the audience manipulation, participants performed a practice session inside the scanner. The alleged goal of this practice session was to test whether the camera connection with the peer was working, which was always positively confirmed after this brief practice. In order to make sure participants were paying attention to the Audience manipulation, we asked participants to indicate by a button press (‘yes’ or ‘no’) after each Audience block whether or not they had been observed (‘Camera rating’). They were told their responses to this question were required for the peer to know which blocks' data to evaluate afterwards.

#### 2.2.3 Block design

Participants performed two sessions of the relational reasoning task, each comprising ten alternating Audience blocks (five Peer and five Alone, Fig. 1(e)). Whether a session started with a Peer or an Alone block was counterbalanced across the two sessions within and between subjects. After every two Audience blocks, there was a fixation baseline block (16 s, 4 per session). Each Audience block lasted 34.4 s and was preceded by a 3 s information screen about the status of the camera, and followed by 3 s window for participants to input whether the camera had been on or off (‘Camera rating’). Within each Audience block, there was one Control block and one Relational block each lasting 16 s, consisting of 4 trials preceded by a 1.2 s Task block instruction screen. Participants equally often started an Audience block with a Relational or a Control block, and this was randomized within a session. Participants had a maximum of 4 s to input their response on each trial, during which time the stimulus was displayed for 3.5 s and followed by a blank screen for 0.5 s.

### 2.3 Data acquisition

Brain imaging data were acquired on a Siemens Avanto 1.5 T MRI scanner (Erlangen, Germany). Structural data were acquired with a T_1_-weighted fast-field echo structural image sequence lasting 5 min 30 s Functional data were acquired in two sessions each lasting 8 min and 6 s with a multi-slice T_2_*- weighted echo-planar sequence with blood-oxygen level dependent (BOLD) contrast (repetition time (TR)=2.975 s, echo time (TE)=0.05 s). In each session, 162 volumes were sampled and each volume comprised 35 axial slices (in-plane resolution: 3×3×3 mm^3^) covering most of the cerebrum. Participants also performed three additional runs, each lasting 6 min, of another task, after the functional and structural data for this task were collected.

The task was presented and responses were acquired with Cogent 2000 (www.vislab.ucl.ac.uk/Cogent/index.html) using Matlab R2010b (Mathwork Inc. Sherborn, MA). Stimuli were front-projected onto a screen, which participants viewed via a mirror mounted on their head coil.

### 2.4 Data analysis

Behavioural data were analysed with SPSS 21 (Armonk, NY: IBM Corp.). Mean accuracy, mean RT (correct trials only) and RT variability (SD) (all trials) were calculated for each participant in each condition. Separate 2×2×2 mixed-design ANOVAs with Audience (Alone; Peer) and Task (Control; Relational) as within-subjects factors and Age group (adolescents; adults) as between-subjects factor were employed to analyse each of these three measures.

Functional imaging data were preprocessed and analysed using SPM8 (Statistical Parametric Mapping, Wellcome Trust Centre for Neuroimaging, http://www.fil.ion.ucl.ac.uk/spm/). To allow for T1 equilibration effects, the first four volumes of each session were discarded. Images were realigned to the first analysed volume with a second-degree B-spline interpolation to correct for movement during the session. The bias-field corrected structural image was coregistered to the mean, realigned functional image and segmented on the basis of Montreal Neurological Institute (MNI)-registered International Consortium for Brain Mapping (ICBM)-tissue probability maps. Resulting spatial normalisation parameters were applied to the realigned images to obtain normalised functional images with a voxel size of 3×3×3 mm^3^, which were smoothed with an 8-mm full width at half maximum (FWHM) Gaussian kernel.

Realignment estimates were used to calculate framewise displacement (FD) for each volume, which is a composite, scalar measure of head motion across the six realignment estimates (Siegel et al., 2013). Volumes with an FD>0.9 mm were censored and excluded from general linear model (GLM) estimation by including a regressor of no interest for each censored volume. Scanning sessions with more than 10% of volumes censored or a root mean square (RMS) movement over the whole session greater than 1.5 mm (1 session for two adolescent participants) were excluded from the analysis. Adolescent and adult participants did not significantly differ in the number of censored volumes (adolescents =2.32±3.99, adults=1.64±3.39; p>0.6), mean RMS movement (adolescents=0.25 mm ±0.09, adults=0.26 mm±0.09; p>0.8) or mean FD (adolescents=0.12 mm±0.03, adults=0.11 mm ±0.05; p>0.6).

Scanning sessions were treated as separate time series and each series was modelled by a set of regressors in the GLM. The GLM included seven box-car regressors (four task conditions, Instructions, Camera-rating, and Fixation) and one event-related regressor for errors per session, which were convolved with a canonical hemodynamic response function. Censored volumes (FD>0.9) were modelled as separate regressors in the GLM. Data were high-pass filtered (128 s). Resulting parameter estimates were used to create four contrasts comparing each of the four task conditions to the fixation baseline. These contrasts were then entered into a random-effects analysis using a Subject x Age Group x Condition flexible factorial design, modelling all three factors as main effects (the Subject factor was included to account for the repeated-measure nature of the data) and an Age Group x Condition interaction. First, we functionally defined voxels which were significantly activated in the Relational>Control contrast (voxel-level p<0.001 uncorrected, cluster-level p<0.05 family wise error [FWE] corrected). Second, we performed voxel-wise ANOVAs to test for a modulation within this relational-integration network by Age group, Audience and/or Task. We tested for regions within the relational-integration network which showed age-related differences in activation during relational integration (Age group x Task interaction). Next, we tested for regions within the relational-integration network that showed a modulation by Audience when collapsing across Age group (main effect of Audience (Peer; Alone) and Audience x Task interaction). To investigate our main research question, we tested for regions in the relational-integration network in which activation was modulated by Audience differently in the two Age groups (Audience x Age group interaction), and whether this was additionally modulated by the Task factor (Audience x Age group x Task interaction). This is an unbiased method as all interaction analyses performed in the relational-integration network are orthogonal to the task main effect (Kriegeskorte et al., 2009). In addition, to test whether the magnitude of the behavioural audience effect was correlated with individual differences in activation in the audience main effect, we performed a voxel-wise two-sample *t*-test including the behavioural audience effect as covariate of interest. Finally, we performed a correlation analysis between RPI scores and individual differences in activation in the audience main effect.

Exploratory whole-brain analyses were performed to investigate possible additional activations showing an Audience x Age group effect outside of the relational-integration network (see Supplementary Materials).

The results were reported if significant at voxel level p<0.001 uncorrected and cluster-level corrected at p_FWE_<0.05 or voxel-level corrected at p_FWE_<0.05. Significant interactions were followed up by extracting the mean signal across all voxels of significant clusters with MarsBar (Brett et al., 2002) and analysing simple effects in SPSS using *t*-tests (with Bonferroni correction for multiple comparisons).

### 2.5 Debriefing

After the study, participants were informed that, in fact, no one had been observing their performance in the scanner. Only participants who had believed that they were being observed by an actual peer were included in the analysis (n=19/23 adolescents, n=14/18 adults).

## 3 Results

### 3.1 Behavioural results

**Fig. 2:**
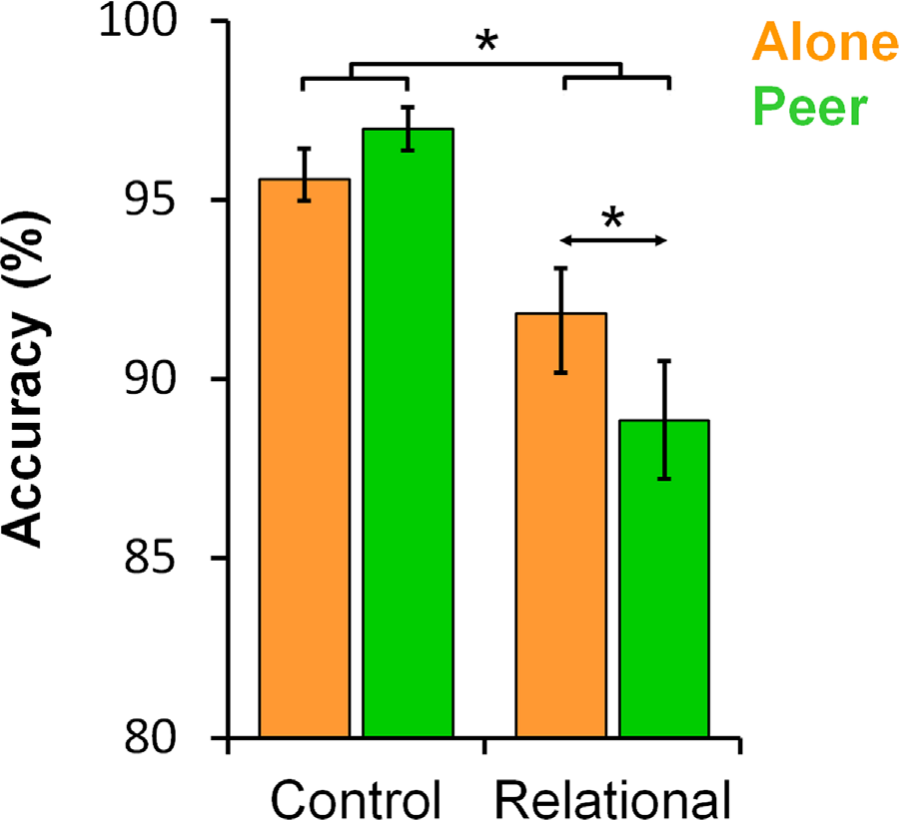
Behavioural audience effect. Accuracy data (mean±SE): There was a significant Audience x Task interaction. Accuracy in the Relational condition in adolescents and adults was reduced in the presence of a peer audience relative to being alone. The increase in accuracy in the Control condition was not significant. The SE is the between-subject SE obtained from the mixed-design ANOVA. ^⁎^ indicates p<0.05.

Accuracy and RT data were analysed with 2 (Audience) x 2 (Task) x 2 (Age group) mixed-design ANOVAs. For *accuracy* (see Table 1), there was a main effect of Task: participants were less accurate in the Relational (mean accuracy: 90.3%±SE: 1.3) relative to the Control condition (96.3%±0.5; F(1,31)=22.98; p<0.001; η_p_^2^=.426, Fig. 2). There was no main effect of either Audience (p>0.2) or Age group (p>0.8). There was a two-way interaction between Task and Audience (F(1,31)=5.98; p=0.020; η_p_^2^=.162, Fig. 2), which was driven by a significant decrease in accuracy in the Peer (88.9%±1.6) relative to the Alone condition for the Relational condition (91.8%±1.3; F(1,31)=6.46; p=0.016; η_p_^2^=.172), but no significant difference between Peer versus Alone for the Control condition (p>0.19). The Audience x Age group interaction (p>0.7) and the three-way interaction between Task, Audience and Age group (p>0.6) were not significant. There was a marginally significant interaction between Task and Age group (F(1,31)=3.12; p=0.087; η_p_^2^=.091).

For the *RT* data (see Table 1), there was a main effect of Task: participants were slower in the Relational (mean RT: 2016 ms±SE: 41) relative to the Control condition (1473 ms±34; F(1,31)=210.27; p<0.001; η_p_^2^=.872). There were no other significant main effects or interactions for the RT data (all ps>0.2). For the *RT variability* data (see Table 1), there was a main effect of Task: participants were less variable in the Control (SD RT: 454 ms±20) relative to the Relational condition (548 ms±20; F(1,31)=37.57; p<0.001; η_p_^2^=.548). The main effects of Age group (p>0.9) and Audience (p>0.3) were not significant. The Task x Age group interaction (p>0.2), the Audience x Age group interaction (p>0.6) and the three-way interaction (p>0.3) were not significant. The interaction between Task and Audience was not quite significant (F(1,31)=3.11; p=0.088; η_p_^2^=.091).

### 3.2 fMRI Results

#### 3.2.1 Definition of the relational-integration network

**Fig. 3:**
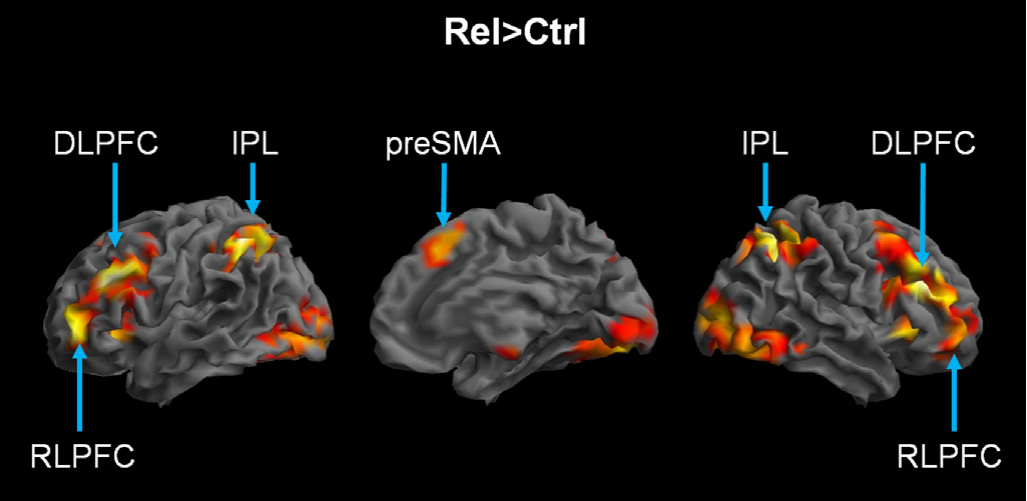
Relational-integration task network. The relational-integration network was defined as the main contrast: Relational>Control (voxel-level uncorrected p<0.001, cluster-level corrected at p_FWE_<0.05) across the average of the two Age groups; the statistical map is rendered on the left, medial and right brain surfaces (left, middle and right panels respectively).

In the first step of analysis, we defined the relational-integration network as the areas activated in the main effect of Task (Relational>Control), combined across age groups. This contrast revealed activations in the preSMA, bilateral inferior and superior parietal lobules including the supramarginal gyrus, bilateral occipital cortex and two large bilateral frontal clusters extending from the middle frontal gyrus, inferior frontal sulcus and inferior frontal gyrus into the RLPFC (Table 2, Fig. 3).

We then performed voxel-wise ANOVAs to test for a modulation within this relational-integration network by Age group, Audience and/or Task.

#### 3.2.2 Developmental changes in relational reasoning activation

**Fig. 4:**
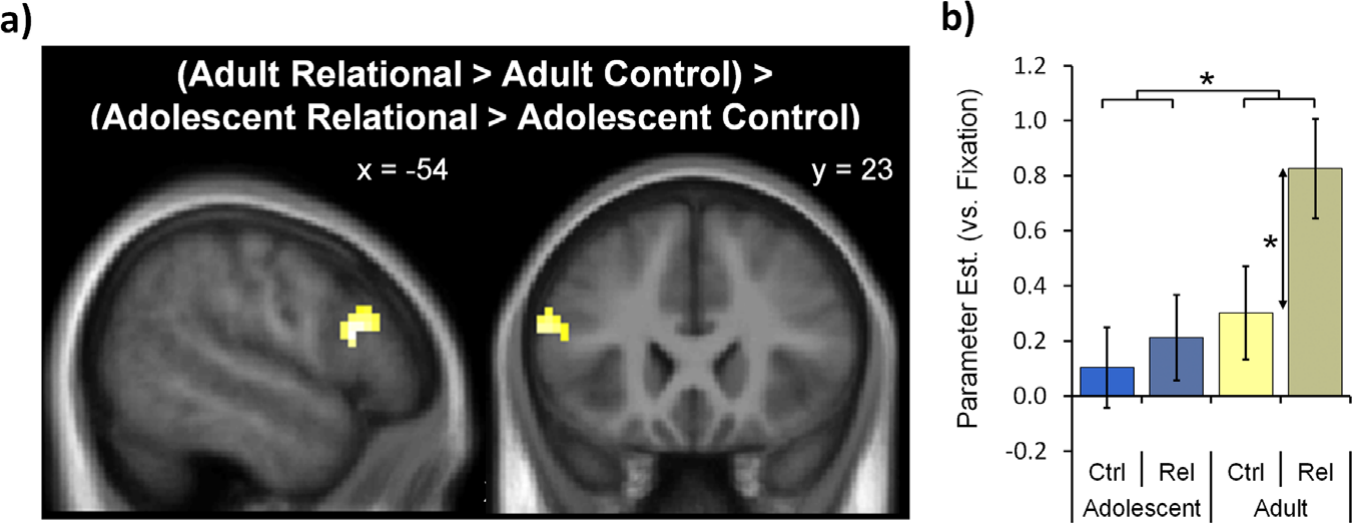
Developmental changes in the main effect of Task. Age differences in Task activation during Relational versus Control within the relational-integration network. a) There was a significant Task x Age group interaction in the left inferior lateral frontal cortex ([−54, 17, 16], voxel-level p_FWE_<0.05, k=43). The cluster is plotted on the average structural brain of the 33 participants. b) In adult participants activation in this left inferior lateral frontal cluster was significantly greater in the Relational compared to the Control condition, while there was no significant difference in adolescents. The bar charts represent mean parameter estimates in the left inferior frontal Task x Age group cluster in the Relational and Control condition plotted against Fixation (mean ± between-subject SE). ^⁎^ indicates p<0.05.

No regions within the relational-integration network showed an Age group x Task interaction with cluster-level correction (p_FWE_<0.05). With voxel-level correction (p_FWE_<0.05), the only difference between Age groups was a cluster in the lateral inferior frontal cortex ([−54,17,16], k=43, Z=4.24, p_FWE_=0.032, Fig. 4): in adults activation in the Relational condition was significantly greater than in the Control condition (t(13)=6.14, p<0.001, η_p_^2^=.133), while there was no significant difference in activation in adolescents (t(18)=1.66, p>0.1, η_p_^2^=.744).

#### 3.2.3 Developmental changes in the audience effect

**Fig. 5:**
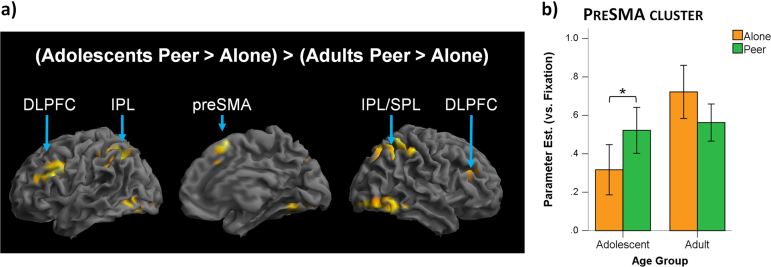
Peer audience effect on relational reasoning task activation. Differential modulation of the activation in the relational-integration network by a peer audience in adolescents and adults. a) The statistical map (voxel-level uncorrected p<0.001, cluster-level corrected p_FWE_<0.05) shows activation demonstrating an Audience x Age group interaction in several regions including: bilateral frontal clusters (inferior and middle frontal cortex), bilateral parietal clusters (inferior and superior parietal cortex), bilateral occipito-temporal clusters and a bilateral preSMA cluster. b) All Age group x Audience clusters showed a consistent activation pattern, exemplified here for the preSMA cluster. Adolescents showed increased recruitment when being observed relative to when alone (pairwise comparisons in the bilateral occipito-temporal, preSMA, right frontal and right parietal clusters survive Bonferroni correction). In contrast, adults showed decreased recruitment (although note this decrease did not survive Bonferroni correction for the multiple regions included in this analysis). The bar chart represents mean parameter estimates of the preSMA Age group x Audience cluster averaging across tasks (Control and Relational) and plotted against Fixation (mean±between-subject SE). ^⁎^ indicates p<0.05.

No regions in the relational-integration network showed a significant modulation of activation by Audience when collapsing across Age group (no main effect of Audience (Peer; Alone) or Audience x Task (Relational; Control) interaction in either direction). However, the analysis of the interaction between Audience and Age group revealed significant bilateral frontal clusters (inferior and middle frontal cortex), bilateral parietal clusters (inferior and superior parietal cortex), bilateral occipital clusters extending into the temporal cortex and a bilateral preSMA cluster (Table 3, Fig. 5). The three-way interaction between Age group, Audience and Task showed no significant clusters, indicating that the Audience by Age group interaction was not further modulated by Task. Paired *t*-tests ran on mean parameter estimates from the nine Audience x Age group clusters showed a consistent pattern in all clusters: adolescents activated these regions more when being observed by the Peer relative to when Alone, while adults showed the reverse effect (note that most of the adolescent effects survived Bonferroni correction for multiple comparisons, while adult effects did not; see Table 3 for pair-wise comparisons statistics). Exploratory whole-brain analyses investigating Audience x Age group effects outside the relational-integration network indicated that additional regions showed a similar pattern of greater activation the Peer>Alone contrast in adolescents compared to adults (see Supplementary Materials). No regions were more active in adults relative to adolescents in the Peer>Alone contrast.

#### 3.2.4 Correlation between BOLD signal and behaviour

In a final series of voxel-wise analyses, we assessed whether individual differences in the neural correlates of the Audience effect were associated with the effect of Audience on accuracy, or individual RPI scores, across the whole sample or within the adolescent or adult group separately. We investigated whether the effect of Audience on performance – i.e. the decrease in accuracy in the Relational condition when the participant was observed versus alone – was correlated with the effect of peer audience on the activation in the Relational versus Fixation contrast. No significant clusters were found in this analysis. In a second analysis, we found there were no significant clusters in the Peer>Alone contrast that correlated with the RPI scores of participants.

## 4 Discussion

In this study, we investigated how peer observation affects behavioural performance and neural activation during a high-level cognitive task in female mid-adolescents and young adults. The peer audience manipulation affected performance in the relational reasoning task, specifically in the condition requiring relational integration: both adolescents and adults showed a decrease in accuracy in the Relational task when being observed by a Peer relative to when Alone. Supporting previous studies (Christoff et al., 2003, Crone et al., 2009, Dumontheil et al., 2010), we found activation in a fronto-parietal network of regions during relational integration (Relational condition) in comparison to when participants had to consider only a single relation (Control condition). Only one region showed a developmental change in relational reasoning activation: adults activated the left inferior lateral PFC in the Relational relative to the Control condition more than did adolescents. Finally, the fMRI analysis revealed that several regions within the relational-integration network were modulated by the peer audience manipulation and that this effect was dependent on the Age group of the participants.

### 4.1 Relational-integration task network

In order to analyse the modulation of the relational-integration network by age and by peer audience, we first functionally defined the regions activated in the Relational condition relative to the Control condition. This contrast revealed a fronto-parietal task network, extending anteriorly into the RLPFC, which is typically and robustly found in fMRI studies of relational integration (Christoff et al., 2003, Crone et al., 2009, Dumontheil et al., 2010, Krawczyk, 2012; Smith et al., 2007, Wendelken et al., 2011).

### 4.2 Developmental changes in the main effect of Task

Previous behavioural and neuroimaging studies have shown that relational reasoning performance as well as the associated neural activation pattern continue to change throughout childhood and adolescence (Crone et al., 2009, Dumontheil, 2014, Dumontheil et al., 2010, McArdle et al., 2002, Wendelken et al., 2011). The current study found no performance changes between mid-adolescence and adulthood, and the only developmental difference in the imaging analysis when contrasting the Relational and Control condition was a cluster in the left inferior lateral PFC, which showed greater BOLD signal increases for relational integration in adults than adolescents. As the focus of this study was the audience effect, we discuss these results in the Supplementary Materials.

### 4.3 Peer audience effect on relational reasoning performance

Previous audience effect studies have found that the presence of an audience generally leads to improvements in performance in simple tasks and impairments in complex tasks (Zajonc, 1965; for a review Bond and Titus, 1983, Guerin, 1986). Consistent with this literature, we found that accuracy in the more complex condition (the relational integration condition) was impaired when participants thought they were being observed by a peer via a camera. However, the increase in accuracy in the simple condition (Control condition) when participants thought they were being observed was not significant. As overall accuracy in this condition was high (>96%), potential improvement might have been masked by a ceiling effect. In a meta-analysis of the social facilitation literature, Bond and Titus (1983) found only a small effect size (Cohen's d=0.11) for an improvement in accuracy (or other qualitative measures) in simple conditions in the presence of others. This small effect size might be due to possible ceiling effects as well as a publication bias towards results that fit with the predicted direction of the social facilitation effect. In addition, inconsistency in the literature in the classification of tasks as simple or complex might explain this small effect size (Bond and Titus, 1983).

In a previous behavioural study, we demonstrated that adolescents’ – particularly mid-adolescents’ – relational reasoning performance was especially sensitive to a physically present peer audience (Wolf et al., 2015). Specifically, mid-adolescents’ relational reasoning performance (accuracy and RT) was compromised when being observed by a peer audience relative to a non-peer audience, while performance in adults was not influenced by the presence of an audience. The current fMRI study differed in a number of ways from this behavioural study, particularly in that it employed a virtual, minimal, unknown peer manipulation, and did not reveal a similar heightened behavioural sensitivity to a virtual peer audience in adolescence. The results instead revealed a comparable decrement in relational integration accuracy in both mid-adolescents and adults when allegedly being observed by a peer audience relative to being alone. Apart from differences in the peer condition (physical presence of a friend versus a minimal, virtual peer manipulation), these differing results may also be attributable to differences in time constraints to solve relational reasoning problems (self-paced versus 4 s), and longer audience sessions (6.6 min) versus shorter audience blocks (34.4 s). In addition, the audience conditions differed with respect to the instructions participants were given regarding the observer. In the previous behavioural study, participants were not given explicit instructions to pay attention to the peer observing them. In contrast, in the current fMRI study, participants were asked to pay attention to the camera light in order to know whether the peer was watching them or not – in other words, participants were explicitly instructed to think about the peer. Despite the fact that the peer was not physically present, this explicit instruction may have increased the salience and impact of the social context in the fMRI study, leading to the behavioural audience effect being observed not only in the adolescents, but also in the adults.

### 4.4 Peer audience effect on the neural correlates of relational reasoning

The majority of experimental studies on the influence of peers on adolescent behaviour have focused on risky and reward-related decision-making (Chein et al., 2011, Gardner and Steinberg, 2005, O’Brien et al., 2011, Reynolds et al., 2013, Smith et al., 2014, Weigard et al., 2014). These studies have suggested that peers influence adolescent risky decision-making by affecting adolescent reward sensitivity and have demonstrated a modulation of activation in reward-related regions in adolescents but not in adults. We were interested in whether adolescent sensitivity to peer influence also extends to differential peer audience effects on other cognitive networks: in particular the neural network of a high-level cognitive task. The fMRI analysis demonstrated that adolescent and adult activations in the relational-integration network were indeed differentially modulated by a peer audience. Several regions within this network – a right inferior frontal cluster, a right parietal cluster, bilateral occipito-temporal clusters and a bilateral preSMA cluster - showed increased activation during relational reasoning when adolescents thought they were being observed compared to when they were alone. These regions showed the opposite pattern in adults, i.e. a decrease in activation, but the adult effects did not survive Bonferroni correction. The Audience-by-Age group interactions were therefore predominantly driven by a significant increase in activation in the presence of a peer audience in adolescents. These findings are in line with our initial prediction that adolescents would show a greater modulation of the activation in the relational-integration network by a peer audience than would adults.

The current study employed a minimal, virtual, but explicit, peer manipulation to investigate the peer audience effect on activation in a high-level cognitive task-network. A similar manipulation has been previously used in a study by Somerville et al. (2013), who found peak levels of embarrassment in late adolescence and greater activation in the mPFC in adolescence and adulthood relative to late childhood, when the participants thought they were being watched. Although it was not the primary focus of this study, we checked for audience effects outside the relational-integration network (see Supplementary Materials). This analysis found no evidence for an audience effect in typical social brain regions (Frith and Frith, 2007), neither when collapsing across age groups nor when contrasting adolescent and adult brain activation patterns. These differing results might be related to the specific peer manipulation and the task that participants performed during the experiment. Although in both studies participants were explicitly asked to monitor when they were being watched, in Somerville et al. (2013), participants believed that their face was observed via camera, while in the current study participants were told that both they and their performance would be observed. In Somerville et al., (2013), participants did not perform a cognitive task beyond monitoring the changing camera status and rating how much they had experienced happiness, excitement, nervousness, worry, fear and embarrassment in the preceding block. Participants therefore likely spent more time thinking about the peer, what the peer thought of them, and their own mental states (mentalising) than the participants in the current study, who performed a speeded reasoning task and were likely to have had fewer cognitive resources available to think about the peer's evaluation or their own mental states.

We can only speculate about the cognitive mechanisms underlying the observed neuroimaging audience effect pattern in our experiment. The increase in activation in the relational-integration network in the presence of a peer audience in adolescents might be associated with attentional distraction by the peer observation or increased arousal. Compared with adults, adolescents might be more preoccupied by what the peer thinks about them and thus show greater attentional distraction. How a distractor might affect task-related activation is still debated. FMRI studies investigating the effect of emotional distractors on task performance have demonstrated both an increase in activation in task-related regions in the presence of emotional distractors (Wessa et al., 2013), and a deactivation in task-related regions (Dolcos and McCarthy, 2006, Mitchell et al., 2008). Our neuroimaging results suggest that adolescents show neural sensitivity to a peer audience in a way that is not specific to task condition and not paralleled by differences in task performance. Note that, as attentional distraction may lead to greater RT variability, we compared RT variability for the Peer and the Alone condition. We found no effect of Audience on RT variability across the whole sample or between age groups. The interpretation that greater activation in the presence of peers may reflect greater attentional distraction in adolescents would need to be investigated in future studies, which could assess whether a non-social distractor evokes similar effects as the presence of a peer audience.

Another theory in the social psychology literature suggests that audience effects might be driven by increased arousal in the presence of an audience (Zajonc, 1965). Adolescents show increased levels of autonomic arousal compared to children and adults, when they think they are being observed by a peer via a camera in the scanner (Somerville et al., 2013). Consequently, differences in the peer audience effect in adolescents and adults might be due to differences in autonomic arousal in the presence of a peer audience. In adults, autonomic arousal was elevated across all levels of a working memory task in the presence of an evaluative, expert audience relative to alone (although this social condition additionally had a competitive component; Ito et al., 2011). In contrast, participants’ performance was impaired only in the most difficult condition (3-back) and NIRS data showed that prefrontal activation was increased in the 3-back condition relative to baseline. Note that NIRS does not allow sufficient spatial resolution to localize whether the increase in activation was within or outside the working memory network. The different patterns of findings of this study suggest that the audience effect on working memory performance might not purely be mediated by arousal (Ito et al., 2011). However, the study differed from ours because it involved an additional competitive component in the audience condition, an adult sample and a non-peer audience. Whether the neural peer audience effect we observed in adolescence is mediated by heightened arousal associated with the presence of a peer could be investigated in future studies.

The behavioural and neuroimaging findings of our study do not directly map onto each other. Notably, although the audience effect on accuracy was observed across age groups and was specific to the Relational condition, the audience effect on brain activation was specific to the adolescents and observed across Control and Relational conditions. Similar incongruencies were found by Somerville et al. (2013) when comparing conditions in which participants were being watched or anticipated being watched compared to when they were not being watched. Quadratic effects of age on embarrassment ratings and skin conductance measures were observed, with a peak in mid- to late adolescence. In contrast, there were plateauing effects of age (differences between children and older participants, but not between adults and adolescents) on mPFC activation and connectivity between the caudate and mPFC. Ito et al. (2011) also observed different patterns of the audience effect on skin conductance, behaviour and NIRS data as a function of task difficulty. Beyond differences in the sensitivity of these methods (e.g. evidenced by greater sensitivity of neuroimaging data than behavioural data to genetic differences; Dumontheil et al., 2011), these differences may be due to the fact that behaviour reflects a large combination of factors beyond the block-related activations measured in the current fMRI paradigm, such as event-related activations, or slower fluctuations in attention and mood over time.

### 4.5 Limitations and implications

The study has a number of limitations, some of which may be addressed by future studies. The current study included female participants only. Future studies should investigate whether similar developmental differences in the audience effects on the neural correlates of relational reasoning can be found in male participants and whether there might be differences in the peer audience effects depending on whether the observing audience is a same-sex or other-sex peer. Due to the relatively large number of participants who did not believe the peer audience manipulation, our final sample (particularly the adult group) is relatively small and further replications are needed.

Previous fMRI and eye-tracking studies have also employed virtual peers in the creation of different peer conditions, in which, for example, participants were led to believe that they were interacting with virtual peers, received online peer feedback or were observed by virtual peers (for example, Guyer et al., 2009, Jones et al., 2014, Moor et al., 2010, Sebastian et al., 2011, Silk et al., 2012, Somerville et al., 2013). Our behavioural and neuroimaging results demonstrate that the peer audience manipulation was successful; however it would be interesting to investigate whether these peer audience effects are dependent on the familiarity and physical presence of the peer. Similarly to previous fMRI studies investigating peer influence effects (Chein et al., 2011; Smith et al., 2014, Somerville et al., 2013), the current study included two levels of the Audience factor, i.e. a Peer versus an Alone condition. This design does not allow the conclusion that the observed audience effects were specific to the presence of peers. Future studies – comparing a Peer to a Non-Peer to an Alone condition – should assess the specificity of this audience effect.

Broadening our knowledge of peer audience effects in adolescence might benefit the design of educational contexts. While it is important that students can learn and work in environments that optimally support their skills acquisition, it is also important that they learn how to excel in tasks while being observed and evaluated by their peers. Our research provides evidence for a differential modulation of a high-level task-network in adolescents and adults by a peer audience. Further studies are required to understand the exact cognitive mechanisms underlying the effect. If adolescents activate compensatory mechanisms in order to maintain their performance level in the presence of a peer audience, educational settings might attempt to balance appropriately between the two types of environment. This would allow adolescent students to become proficient in the task itself, and to learn to carry out tasks in the presence of evaluative peers.

## Figures and Tables

**Table 1:** Accuracy, RT and RT variability data in the relational reasoning task (mean and SD for the four conditions and the two age groups).

		**Control Alone**		**Relational Alone**		**Control Peer**		**Relational Peer**	
		**Mean**	**SD**	**Mean**	**SD**	**Mean**	**SD**	**Mean**	**SD**
**Accuracy (%)**	**Adolescents**	94.74	5.71	92.76	6.34	95.92	3.56	90.39	7.87
**Adults**	96.43	3.06	90.89	8.06	98.04	3.28	87.32	11.03
									
**RT (ms)**	**Adolescents**	1453	247	1938	245	1481	208	1995	257
**Adults**	1498	149	2055	195	1460	241	2075	248
									
**RT variability (ms)**	**Adolescents**	443	125	542	121	450	146	572	165
**Adults**	479	99	515	88	446	127	561	110

**Table 2:** Relational-integration network. Main effect of Task (Relational>Control; voxel-level uncorrected p<0.001, cluster-level corrected at p_FWE_<0.05).

**Cluster**	**Brain region**	**Size (N voxels)**	**Z**	**Peak voxel (in mm)**		
**x**	**y**	**z**
**Left frontal**	**Inferior frontal gyrus (DLPFC)**	**1344**	**>8**	**−48**	**26**	**28**
	Middle frontal gyrus (RLPFC)		7.59	−48	50	−5
	Middle frontal gyrus (RLPFC)		6.53	−39	53	7
**Left parietal**	**Inferior parietal lobule (IPL)**	**750**	**7.71**	**−45**	**−49**	**55**
	Inferior parietal lobule (IPL)		7.30	−42	−43	40
**Right frontal**	**Inferior frontal gyrus (DLPFC)**	**1621**	**7.57**	**45**	**32**	**25**
	Anterior insula		6.58	33	23	−2
	Superior frontal gyrus		6.14	21	44	−11
**Bilateral occipital**	**Lingual gyrus**	**2441**	**7.42**	**21**	**−85**	**−5**
	Middle occipital gyrus		7.02	−18	−91	−2
	Inferior occipital gyrus		6.54	45	−70	−17
**Right parietal**	**Superior parietal lobule**	**963**	**7.18**	**39**	**−55**	**55**
	Angular gyrus		6.96	33	−58	43
	Inferior parietal lobule (IPL)		6.86	42	−43	43
**Bilateral preSMA**	**PreSMA**	**324**	**6.38**	**−3**	**17**	**52**

**Table 3:** Developmental changes in the effect of Audience in the relational-integration network. Regions within the relational-integration network showing an Audience x Age group interaction (contrast: [(Adolescent-Peer>Adolescent-Alone)>(Adult-Peer>Adult-Alone)]; voxel-level uncorrected p<0.001, cluster-level corrected p_FWE_<0.05 or ^(a)^ voxel-level corrected p_FWE_<0.05). The columns on the far right provide the p-values and effect sizes for the pairwise comparisons of Peer versus Alone in each Age group using the mean parameter estimates of each cluster (^(b)^ survive Bonferroni correction).

**Cluster**	**Brain region**	**Size (N voxels)**	**Z**	**Peak voxel (in mm)**			**Adolescent pairwise comparison (Peer versus Alone)**		**Adult pairwise comparison (Peer versus Alone)**	
				**x**	**y**	**z**	**p-value**	**η**_**p**_^**2**^	**p-value**	**η**_**p**_^**2**^
**Left parietal**	Inferior parietal lobule	**408**	**5.15**	**−33**	**−52**	**43**	0.004	0.384	0.004	0.476
	Superior occipital cortex		4.23	−24	−67	28				
	Superior parietal lobule		4.08	−15	−64	49				
**Left frontal**	Inferior frontal gyrus (pars opercularis)	**307**	**4.92**	**−39**	**11**	**28**	0.003	0.392	0.003	0.515
	Middle frontal gyrus		4.26	−45	35	19				
**Right parietal**	Superior parietal lobule	**424**	**4.68**	**42**	**−49**	**61**	0.002^(b)^	0.414	0.025	0.329
	Inferior parietal lobule		4.10	36	−52	46				
	Precuneus		3.96	12	−67	49				
**Bilateral preSMA**	PreSMA	**184**	**4.52**	**3**	**14**	**58**	0.001^(b)^	0.479	0.012	0.394
	Medial superior frontal gyrus		3.76	−6	23	37				
	Medial superior frontal gyrus		3.61	3	26	37				
**Right occipito-temporal**	Inferior temporal gyrus	**262**	**4.49**	**51**	**−55**	**−20**	0.002^(b)^	0.437	0.043	0.279
	Inferior occipital gyrus		4.16	39	−85	−14				
	Fusiform gyrus		3.98	27	−61	−14				
**Left occipito-temporal**	Fusiform gyrus	**126**	**4.47**	**−27**	**−64**	**−17**	0.002^(b)^	0.002	0.028	0.028
	Inferior temporal gyrus		4.25	−45	−61	−11				
	Inferior occipital gyrus		3.86	−45	−73	−14				
**Left occipital**	Lingual gyrus	**50**	**3.96**	**−18**	**−82**	**1**	0.008	0.335	0.023	0.337
	Superior occipital gyrus		3.42	−9	−97	4				
	Lingual gyrus		3.16	−12	−91	−11				
**Right frontal**	Inferior frontal gyrus (pars triangularis)	**54**	**3.78**	**42**	**29**	**28**	0.001^(b)^	0.483	0.014	0.385
**Left orbitofrontal**^**(a)**^	Inferior frontal gyrus (pars orbitalis)	**23**	**4.39**	**−30**	**29**	**−5**	0.003	0.394	0.008	0.434

## Footnotes

Appendix A Supplementary data associated with this article can be found in the online version at 10.1016/j.neuropsychologia.2016.05.001.
