# Supplementary material for: Audience effects on the neural correlates of relational reasoning in adolescence
Source: Neuropsychologia. 2016 Jul 1;87:85–95. doi: 10.1016/j.neuropsychologia.2016.05.001 (PMC4915335; doi:10.1016/j.neuropsychologia.2016.05.001)
Supplement: Supplementary file 1 — Supplementary material [file mmc1.docx]

**FMRI whole-brain analyses**

**Whole-brain analysis of developmental changes in relational reasoning activation**

No regions showed an Age group x Task interaction in the whole-brain analysis.

**Whole-brain analysis of the audience effect**

In order to investigate the modulation of activation in a high-level cognitive task-network by an audience, the analysis of the audience effect in the main analysis was restricted to the relational-integration network. For completeness, we also report here the analysis of the effect of Audience from the whole-brain analysis. No region showed a significant modulation of activation by Audience when collapsing across Age group (no main effect of Audience or Task x Audience interaction in either direction). The analysis of the interaction between Audience and Age group revealed significant left frontal clusters (inferior and middle frontal cortex), a bilateral preSMA cluster, a large bilateral posterior cluster extending into the temporal, parietal and occipital cortex, a right occipito-temporal cluster, a bilateral caudal cluster and a cluster in the right putamen (Supplementary table 1, Supplementary figure 1). Paired t-tests ran on mean parameter estimates from the seven Audience x Age group clusters showed the same pattern as in the main analysis: greater activation in the Peer condition relative to the Alone condition in adolescent participants and the reverse effect for adults (see Supplementary table 1 for pair-wise comparison statistics). There were no regions that were more activated in adults relative to adolescents in the Peer>Alone contrast and there was no significant three-way interaction between Age group, Audience and Task.


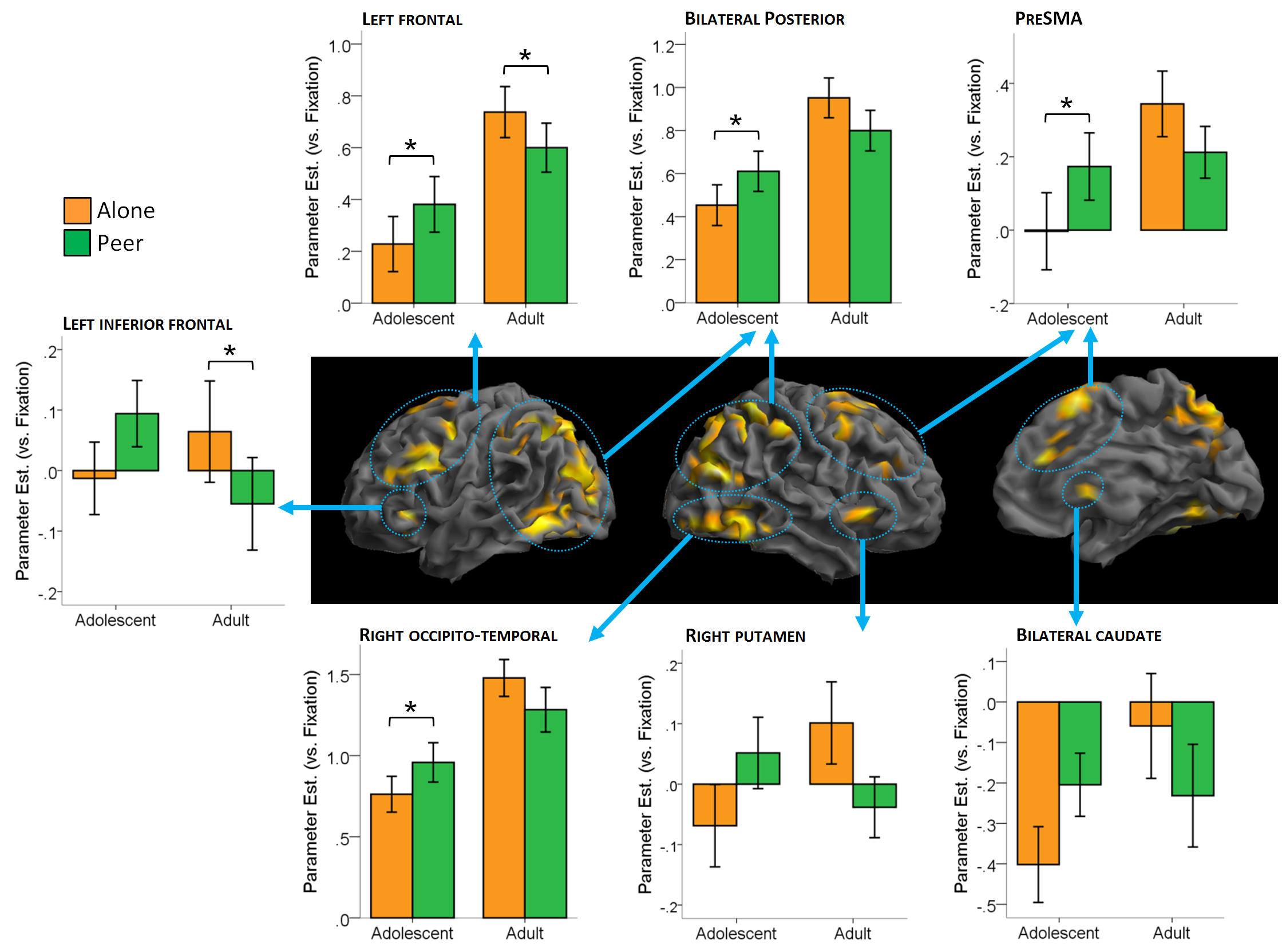


**Supplementary figure 1: Developmental changes in the effect of Audience (whole-brain analysis).** The statistical map (voxel-level uncorrected p < 0.001, cluster-level corrected at p_FWE_ < 0.05) shows activation demonstrating an Audience x Age group interaction in several regions including: left frontal clusters (inferior and middle frontal cortex), a preSMA cluster, a large bilateral posterior cluster extending into the temporal, parietal and occipital cortex, a right occipito-temporal cluster, a bilateral caudal cluster and a cluster in the right putamen. All Age group x Audience clusters showed a consistent activation pattern. Activation was greater when being observed relative to when alone in adolescents, while adults showed the reverse pattern. The bar charts show mean parameter estimates for the Age group x Audience cluster of the Relational and Control tasks combined against Fixation (mean ± between-subject SE). * indicates pairwise comparisons surviving Bonferroni correction.

Supplementary table 1: **Developmental changes in the effect of Audience (whole-brain analysis).** Regions showing an Audience x Age group interaction (contrast: [(Adolescent-Peer>Adolescent-Alone) > (Adult-Peer>Adult-Alone)]; voxel-level uncorrected p < 0.001, cluster-level corrected at p_FWE_ < 0.05). The columns on the far right provide the p-values for the pairwise comparisons of Peer vs. Alone in each Age group using the mean parameter estimates of each cluster (^(^**^a^**^)^ survives Bonferroni correction).

| **Cluster** | **Brain region** | **Size (N voxels)** | **Z** | **Peak voxel (in mm)** | | | **Adolescent pairwise comparison (Peer versus Alone)** | | **Adult pairwise comparison (Peer versus Alone** | |
| --- | --- | --- | --- | --- | --- | --- | --- | --- | --- | --- |
|  |  |  |  | **x** | **y** | **z** | **p-value** | **η_p_^2^** | **p-value** | **η_p_^2^** |
| **Bilateral posterior** | **Inferior parietal lobule** | **3027** | **5.15** | **-33** | **-52** | **43** | **0.001^a^** | **.478** | **0.007** | **.443** |
|  | Middle temporal gyrus |  | 4.95 | 48 | -70 | 22 |  |  |  |  |
|  | Middle occipital gyrus |  | 4.91 | -33 | -82 | 25 |  |  |  |  |
| **Left frontal** | **Inferior frontal gyrus (pars opercularis)** | **498** | **4.92** | **-39** | **11** | **28** | **0.002^a^** | **.428** | **0.001^a^** | **.553** |
|  | Precentral gyrus |  | 4.39 | -27 | -4 | 46 |  |  |  |  |
|  | Middle frontal gyrus |  | 4.26 | -45 | 35 | 19 |  |  |  |  |
| **Left inferior frontal** | **Inferior frontal gyrus** | **94** | **4.68** | **-27** | **29** | **-8** | **0.006** | **.349** | **0.003^a^** | **.512** |
|  | **(pars orbitalis)** |  |  |  |  |  |  |  |  |  |
|  | Putamen |  | 3.16 | -27 | 14 | -8 |  |  |  |  |
| **Right occipito-temporal** | **Inferior temporal gyrus** | **404** | **4.58** | **51** | **-52** | **-20** | **0.001^a^** | **.478** | **0.030** | **.312** |
|  | Inferior occipital gyrus |  | 4.16 | 39 | -85 | -14 |  |  |  |  |
|  | Lingual gyrus |  | 4.00 | 15 | -61 | -8 |  |  |  |  |
| **PreSMA** | **PreSMA** | **728** | **4.52** | **3** | **14** | **58** | **<0.001^a^** | **.505** | **0.004** | **.493** |
|  | Middle frontal gyrus |  | 4.37 | 27 | 11 | 43 |  |  |  |  |
|  | Middle frontal gyrus |  | 4.16 | 24 | 41 | 25 |  |  |  |  |
| **Right putamen** | **Putamen** | **76** | **4.26** | **27** | **17** | **-5** | **0.005** | **.360** | **0.005** | **.471** |
|  | Insula |  | 4.00 | 36 | 17 | -5 |  |  |  |  |
|  | Insula |  | 3.22 | 39 | 11 | -17 |  |  |  |  |
| **Bilateral Caudate** | **Caudate** | **108** | **4.23** | **3** | **8** | **1** | **0.008** | **.333** | **0.025** | **.329** |
|  | Caudate |  | 3.96 | -9 | 17 | 7 |  |  |  |  |
|  | Caudate |  | 3.51 | -9 | 2 | 7 |  |  |  |  |

**Discussion of the developmental changes in the main effect of Task**

In the current study, we employed a relational reasoning paradigm (Christoff, Ream, Geddes, & Gabrieli, 2003; Smith, Keramatian, & Christoff, 2007) that has previously been used to investigate the development of neural activation associated with relational reasoning (Dumontheil, Houlton, Christoff, & Blakemore, 2010; Wendelken, O’Hare, Whitaker, Ferrer, & Bunge, 2011). These and other developmental studies reported improvement in relational integration abilities between late childhood (8-12 years) and adulthood (Crone et al., 2009) and within late childhood and adolescence (7-18 years, Wendelken et al., 2011). An analysis of longitudinal data demonstrated that improvements in fluid reasoning abilities are greatest in childhood and become smaller in adolescence (McArdle, Ferrer-Caja, Hamagami, & Woodcock, 2002). Improvements between mid-adolescence and adulthood are more difficult to detect, especially when the task only requires the integration of a maximum of two dimensions and smaller samples of participants are employed (Dumontheil et al., 2010, Experiment 2). Consistent with these previous results, the current study did not find significant developmental changes in performance between mid-adolescence and early adulthood.

Neuroimaging studies have reported developmental changes in the activation pattern of the fronto-parietal network involved in relational integration (for a review Dumontheil, 2014). One study demonstrated increasing specificity with age in left RLPFC and bilateral IPL activation for relational integration in a group of 7- to 18-year-olds (Wendelken et al., 2011). Another study reported, in a group of young adolescents (11-14 years), mid-adolescents (14-18 years) and adults (22-30 years), an increase in activation in the left RLPFC during the relational relative to the control task between young adolescence and mid-adolescence, followed by a decrease in activation between mid-adolescence and adulthood, with a similar decrease in activation in the left anterior insula (Dumontheil et al., 2010). Activation in the preSMA decreased between young adolescence and adulthood. Here, as a first step in our analysis, we investigated general developmental differences in activation associated with relational integration. The only region that showed a developmental difference during relational reasoning was the left inferior lateral PFC, with greater activation in adults relative to adolescents during relational integration compared to the manipulation of single relations. Neuroimaging results of relational reasoning tasks have been interpreted as showing that activation in the relational-integration network, in particular the RLPFC, becomes increasingly specific during childhood and adolescence to trials that require the consideration of more relative to fewer dimensions (Dumontheil et al., 2010; Wendelken et al., 2011, Dumontheil, 2014). The developmental pattern in the inferior lateral PFC observed in this study might reflect a similar increase in specificity. The previously reported changes in RLPFC and parietal cortex activations with age (Dumontheil et al., 2010; Wendelken et al., 2011) were not observed in this study. This difference may be due to differences in the age distribution of participants, and the more stringent statistical level of significance used for our voxel-wise analyses (cluster-wise or voxel-wise p_FWE_<0.05) compared to the ROI approaches used by Dumontheil et al. (2010) and Wendelken et al. (2011).

**References**

Christoff, K., Ream, J. M., Geddes, L. P. T., & Gabrieli, J. D. E. (2003). Evaluating Self-Generated Information: Anterior Prefrontal Contributions to Human Cognition. *Behavioral Neuroscience*, *117*(6), 1161–1168. http://doi.org/10.1037/0735-7044.117.6.1161

Crone, E. A., Wendelken, C., van Leijenhorst, L., Honomichl, R. D., Christoff, K., & Bunge, S. A. (2009). Neurocognitive development of relational reasoning. *Developmental Science*, *12*(1), 55–66. http://doi.org/10.1111/j.1467-7687.2008.00743.x

Dumontheil, I. (2014). Development of abstract thinking during childhood and adolescence: The role of rostrolateral prefrontal cortex. *Developmental Cognitive Neuroscience*, *10*, 57–76. http://doi.org/10.1016/j.dcn.2014.07.009

Dumontheil, I., Houlton, R., Christoff, K., & Blakemore, S.-J. (2010). Development of relational reasoning during adolescence. *Developmental Science*, *13*(6), F15–24. http://doi.org/10.1111/j.1467-7687.2010.01014.x

McArdle, J. J., Ferrer-Caja, E., Hamagami, F., & Woodcock, R. W. (2002). Comparative longitudinal structural analyses of the growth and decline of multiple intellectual abilities over the life span. *Developmental Psychology*, *38*(1), 115–142. http://doi.org/10.1037/0012-1649.38.1.115

Smith, R., Keramatian, K., & Christoff, K. (2007). Localizing the rostrolateral prefrontal cortex at the individual level. *NeuroImage*, *36*(4), 1387–1396. http://doi.org/10.1016/j.neuroimage.2007.04.032

Wendelken, C., O’Hare, E. D., Whitaker, K. J., Ferrer, E., & Bunge, S. A. (2011). Increased Functional Selectivity over Development in Rostrolateral Prefrontal Cortex. *The Journal of Neuroscience*, *31*(47), 17260–17268. http://doi.org/10.1523/JNEUROSCI.1193-10.2011
